# A phase I study of indoximod in patients with advanced malignancies

**DOI:** 10.18632/oncotarget.8216

**Published:** 2016-03-20

**Authors:** Hatem H. Soliman, Susan E. Minton, Hyo Sook Han, Roohi Ismail-Khan, Anthony Neuger, Fatema Khambati, David Noyes, Richard Lush, Alberto A. Chiappori, John D. Roberts, Charles Link, Nicholas N. Vahanian, Mario Mautino, Howard Streicher, Daniel M. Sullivan, Scott J. Antonia

**Affiliations:** ^1^ H. Lee Moffitt Cancer Center and Research Institute, Tampa, Florida, USA; ^2^ Massey Cancer Center, Virginia Commonwealth University, Richmond, Virginia, USA; ^3^ NewLink Genetics Inc., Ames, Iowa, USA; ^4^ Cancer Therapeutics Evaluation Program, National Cancer Institute, Bethesda, Maryland, USA

**Keywords:** indoleamine 2, 3 dioxygenase, 1-methyl-D-tryptophan, indoximod, immunomodulator

## Abstract

**Purpose:**

Indoximod is an oral inhibitor of the indoleamine 2,3-dioxygenase pathway, which causes tumor-mediated immunosuppression. Primary endpoints were maximum tolerated dose (MTD) and toxicity for indoximod in patients with advanced solid tumors. Secondary endpoints included response rates, pharmacokinetics, and immune correlates.

**Experimental Design:**

Our 3+3 phase I trial comprised 10 dose levels (200, 300, 400, 600, and 800 mg once/day; 600, 800, 1200, 1600, and 2000 mg twice/day). Inclusion criteria were measurable metastatic solid malignancy, age ≥18 years, and adequate organ/marrow function. Exclusion criteria were chemotherapy ≤ 3 weeks prior, untreated brain metastases, autoimmune disease, or malabsorption.

**Results:**

In 48 patients, MTD was not reached at 2000 mg twice/day. At 200 mg once/day, 3 patients previously treated with checkpoint inhibitors developed hypophysitis. Five patients showed stable disease >6 months. Indoximod plasma AUC and Cmax plateaued above 1200mg. Cmax (∼12 μM at 2000 mg twice/day) occurred at 2.9 hours, and half-life was 10.5 hours. C reactive protein (CRP) levels increased across multiple dose levels.

**Conclusions:**

Indoximod was safe at doses up to 2000 mg orally twice/day. Best response was stable disease >6 months in 5 patients. Induction of hypophysitis, increased tumor antigen autoantibodies and CRP levels were observed.

## INTRODUCTION

The main physiologic function of indoleamine 2,3 dioxygenase (IDO) is the breakdown of the essential amino acid tryptophan into various active metabolites such as kynurenine and NAD+. IDO is expressed both by various malignant and normal cells in an inducible fashion upon exposure to γ-interferon. [1-5]

The role of IDO in inhibiting the immune response was first described by Andrew Mellor and David Munn in 1998, showing it prevented rejection of the allogeneic fetus in pregnant mice. [6] High levels of tumor IDO expression cause infiltrating effector T cells to arrest in G1, become anergic, and die by apoptosis. [7, 8] Additionally, toxic metabolites of tryptophan can directly suppress tumor-infiltrating T cells. [9] Finally, IDO expressing dendritic cells can induce systemic anergy by expanding proliferation of T regulatory cells. [10, 11]

The indole-containing compound 1-methyl-DL-tryptophan was identified as a competitive inhibitor of IDO in 1991 [12]. Preclinical data demonstrated the activity of 1-methyl-DL-tryptophan in preventing T cell anergy in tumor-draining lymph nodes, delaying growth of transplanted Lewis lung cancer mouse xenografts, and working synergistically with various chemotherapeutic agents (doxorubicin, cyclophosphamide, paclitaxel) in regression of autochthonous breast tumors in MMTV-Neu mice. [5, 13] In humans, elevated IDO expression in colon and ovarian cancer is associated with a poorer prognosis. [14,15]

This led to the clinical development of 1-methyl-tryptophan. A preclinical study determined that 1-methyl-D-tryptophan (indoximod) had superior *in vivo* antitumor activity when combined with cyclophosphamide so it was selected as the lead compound. [16] Subsequent work indicated that indoximod functions as a tryptophan mimetic that suppresses the downstream effects of IDO activation on amino acid-sensing pathways and mammalian target of rapamycin (mTOR) signaling. [17] Here, we conducted a phase I trial (ClinicalTrials.gov NCI8045/NCT00567931) to evaluate the safety, dosing, pharmacokinetics, and immunologic effects of indoximod.

## RESULTS

### Patient flow and patient population

Table 1 shows the number of patients accrued to each dose level. Of 53 patients screened for the study, 5 did not meet all eligibility criteria. Table 2 describes the demographics, performance status, and tumor types of the 48 patients treated on study.

**Table 1:** Accrual to the various dose levels

Dose Level	Dose of Indoximod	Schedule	No. of Patients (*N*= 48)
1	200 mg	Daily	10
2	300 mg	Daily	4
3	400 mg	Daily	3
4	600 mg	Daily	4
5	800 mg	Daily	5
6	600 mg	Twice/day	6
7	800 mg	Twice/day	4
8	1200 mg	Twice/day	3
9	1600 mg	Twice/day	3
10	2000 mg	Twice/day	6

**Table 2:** Demographics of treated patients

**Demographic**	
**Sex, n (%)**	
Men	23 (48)
Women	25 (52)
**Age, years**	
Median (range)	60 (21-83)
**Race/Ethnicity, n (%)**	
White	38 (80)
Black	5 (10)
Hispanic	3 (6)
Native American	2 (4)
**Disease type, n (%)**	
Sarcoma	11 (23)
NSCLC	10 (21)
Colorectal	8 (17)
Melanoma	5 (11)
Breast	2 (4)
Ovarian	2 (4)
Pancreatic	2 (4)
Unknown primary	2 (4)
Esophageal	1 (2)
Cervical	1 (2)
Liver	1 (2)
Osteosarcoma	1 (2)
Prostate	1 (2)
Uterine	1 (2)
**ECOG performance status, n (%)**	
0	0 (0)
1	34 (70)
2	14 (30)
**Median prior lines of chemotherapy (range)**	
Chemotherapy	3 (1-8)
**Previous therapy, n (%)**	
Chemotherapy	47 (98)
Radiation	19 (39)
Hormonal/endocrine	3 (6)
Biologics/vaccines	21 (43)

### Adverse events

Table 3 lists the frequency of any grade adverse event that occurred regardless of attribution. The most common adverse events were fatigue (56.3%), anemia (37.5%), anorexia (37.5%), dyspnea (35.4%), cough (33.3%), and nausea (29.2%). The majority of events were grades 1 and 2. There were 2 deaths reported within 30 days of last study treatment due to progression of disease. There were no deaths on study treatment. Serious adverse events included central nervous system ischemia, hypophysitis, fracture, bowel obstruction, urinary tract infection, encephalomyelitis, ileus, pain, pleural effusion, confusion, and weakness. There did not appear to be an increase in toxicity with increased doses of indoximod. All treatment discontinuations were due to disease progression and not attributed to toxicities.

**Table 3:** Adverse events by frequency and grade in entire study population (N = 48)

Adverse Event	Grade					Total (*N*)	%
	1	2	3	4	5		
Fatigue	12	13	2			27	56.3
Anemia	9	6	3			18	37.5
Anorexia	16	2				18	37.5
Dyspnea	9	5	1	1	1	17	35.4
Cough	14	2				16	33.3
Nausea	14					14	29.2
Hyponatremia	12					12	25
Hyperglycemia	8	2	1			11	22.9
Headache	8	2				10	20.8
Lymphopenia	6	1	3			10	20.8
Pain abdomen	8	1		1		10	20.8
Pain back	8	2				10	20.8
Sensory neuropathy	8	2				10	20.8
Diarrhea	8		1			9	18.8
Constipation	4	3				7	14.6
Elevated alkaline phosphatase	4	1	2			7	14.6
Rash	5	2				7	14.6
Dehydration	3	2	1			6	12.5
Elevated creatinine	6					6	12.5
Thrombocytopenia	6					6	12.5
Vomiting	6					6	12.5
Edema peripheral	3	1	1			5	10.4
Neutropenia	5					5	10.4
Pain shoulder	3	2				5	10.4
Fever	3	1				4	8.3
Hyperkalemia	4					4	8.3
Light sensitivity	4					4	8.3
Pain leg	2	2				4	8.3
Proteinuria	3	1				4	8.3
Dyspnea on exertion	3	1				4	8.3
Dizziness	1	1	1			3	6.3
Elevated bun	3					3	6.3
Hypocalcemia	1	2				3	6.3
Hypokalemia	2		1			3	6.3
Elevated prothrombin time	2		1			3	6.3
Insomnia	3					3	6.3
Leukocytosis	3					3	6.3
Pain hip	1	2				3	6.3
Pain knee	3					3	6.3
Tingling extremities	3					3	6.3
Urine color change	1	2				3	6.3
Vision blurred	3					3	6.3
Weakness	2		1			3	6.3
Weight loss	3					3	6.3
Urinary tract infection		2	1			3	6.3
Hypophysitis		3				3	6.3

### Dose-limiting toxicities

There were 3 cases of hypophysitis at dose level 1 (all in patients who had received prior checkpoint inhibitor therapy). No additional cases of hypophysitis were confirmed once patients who received prior checkpoint inhibitor therapy were excluded. Two of the patients were melanoma patients who had prolonged stabilization of disease and were able to stay on indoximod while getting endocrine replacement therapy for hypopituitarism. At dose level 6, one patient had a grade 3 urinary tract infection. However, only hypophysitis was attributed to indoximod. All other SAEs were attributable to other causes such as disease progression or other concomitant illnesses. No MTD up to 2000 mg twice daily was identified.

### Response rate

**Figure 1:**
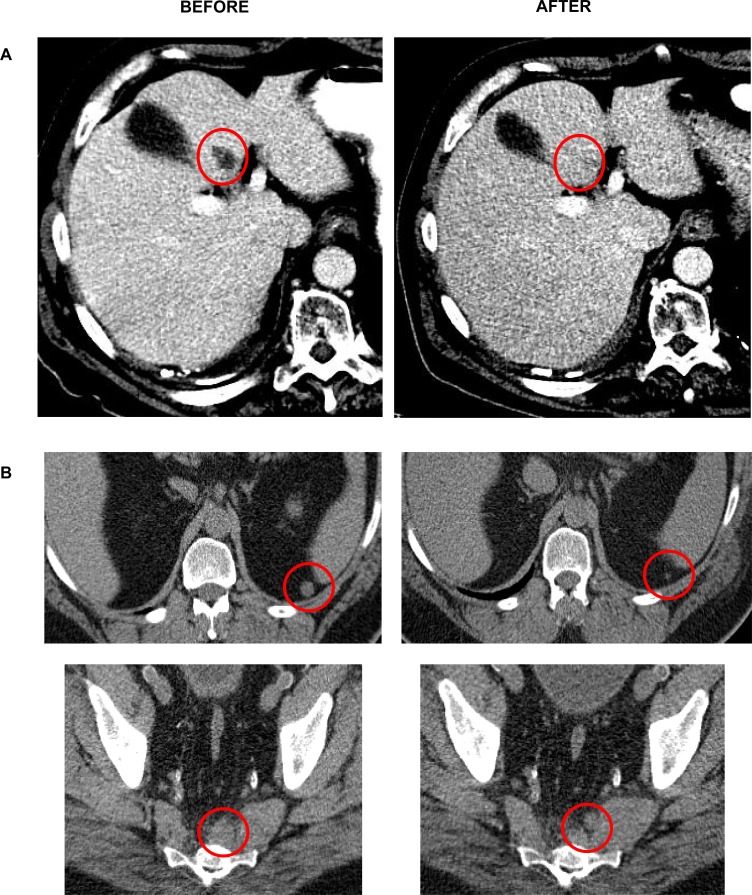
Mixed responses noted in a melanoma (A) and sarcoma (B) patient

The median number of cycles administered was 2 (range 1-8). The best response by site radiologist assessment was stable disease in 5 patients at >6 months duration. These occurred in 2 melanoma patients at dose level 1, 1 colon cancer patient at dose level 2, and 2 sarcoma patients at dose levels 3 and 6. There were also occasional mixed responses noted with resolution of some lesions and emergence of new ones in the same patient (Figure 1).

### Pharmacokinetics and pharmacodynamics

**Figure 2:**
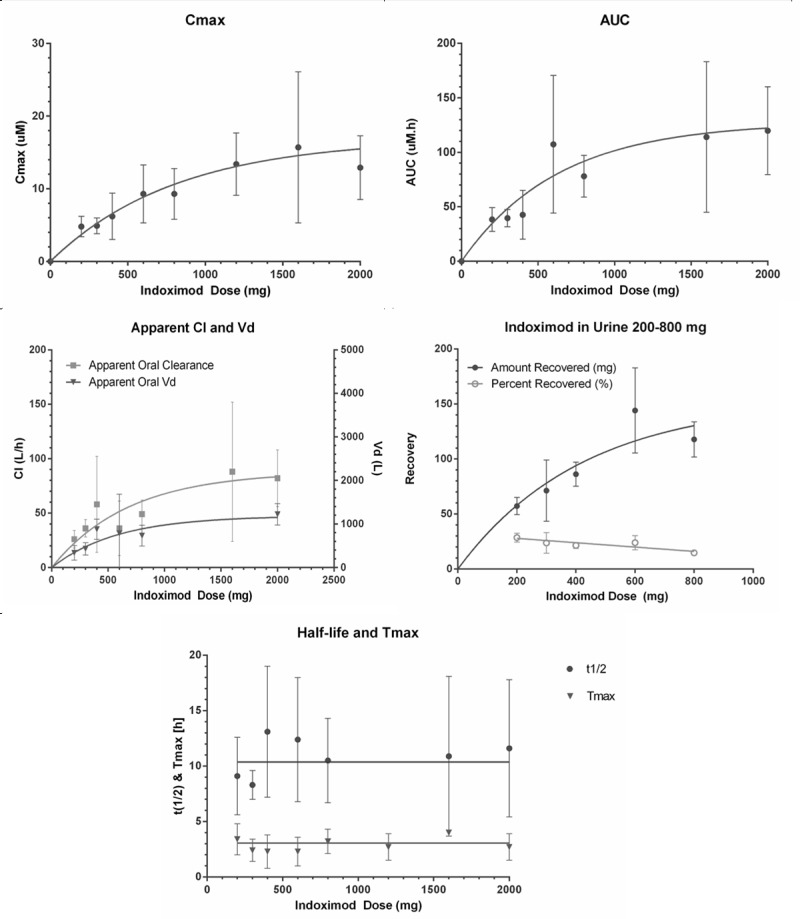
Pharmacokinetics of indoximod

**Figure 3:**
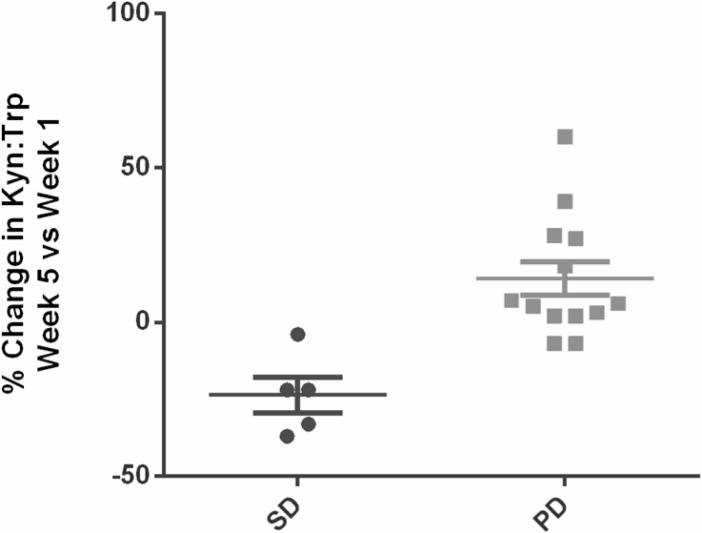
Comparison of percent change in kynurenine-to-tryptophan ratios over 5 weeks on treatment between patients with stable disease versus progressive disease There appears to be a trend toward either no increase or a slight decrease in patients who had prolonged stable disease for over 6 months.

Indoximod demonstrated good oral bioavailability with a linear dose response of AUC and Cmax at up to 800 mg once daily. Beyond this dose, some saturation effects emerged, with plateauing of peak serum levels at single doses at or above 1600 mg. The anticipated therapeutic dose of 10 to 15 μM was reached, suggesting feasibility of current dosing schedules. The half-life of the drug was approximately 10.5 hours, with 20% of the drug renally cleared. Figure 2 graphically summarizes the pharmacokinetic data. The pharmacodynamic data using kynurenine/tryptophan plasma levels at baseline and on treatment for all treated patients did not show a significant change in kynurenine-to-tryptophan ratios at any dose level. This suggests that indoximod does not exert a consistent direct inhibitory effect on IDO enzymatic activity that can be reflected by systemic levels of kynurenine. An interesting observation that will require additional study is that there was a trend toward lower kynurenine-to-tryptophan ratios during treatment in the 5 patients with stable disease when compared with those with progressive disease (Figure 3).

### Immune correlates

**Figure 4:**
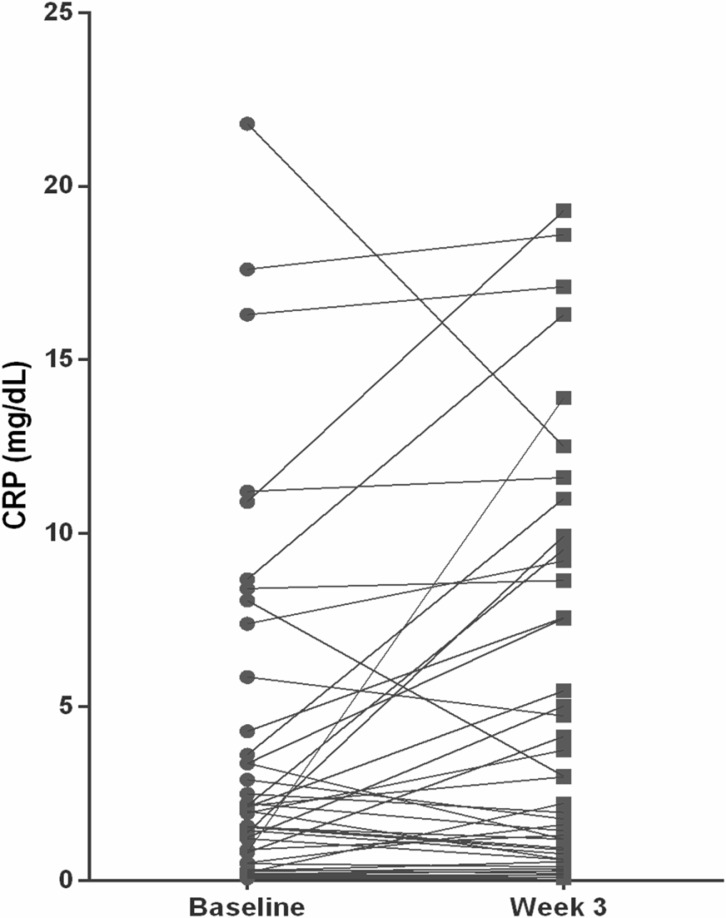
Scatter plot showing the relative changes (in percent) in C-reactive protein at week 3 when compared to baseline The difference between the means (from 3.81 mg/dL at baseline to 5.13 mg/dL at week 3) was statistically significant (*P* = 0.02).

**Figure 5:**
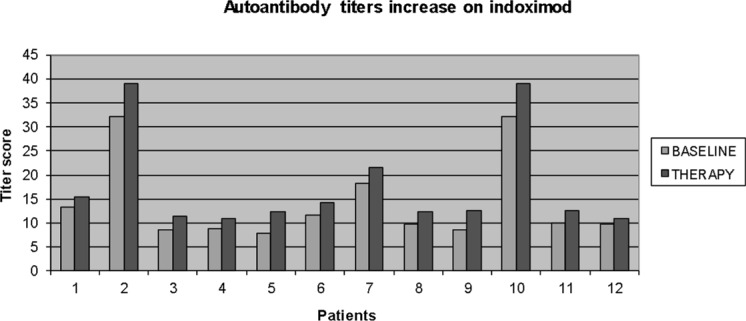
Increases in multiple autoantibody titers were noted in 12 of 40 patients evaluated (including all 3 patients with hypophysitis) at week 5 The differences were statistically significant using a two-tailed *t* test (*P* < 0.05).

The T-cell flow cytometry panel did not identify any statistically significant changes in any of the aforementioned T cell populations from baseline through week 7 across the group as a whole. There also were no detectable dose-dependent changes noted in patients at higher dose levels versus those at lower dose levels. The mean levels of C-reactive protein significantly increased from a baseline level of 3.81 mg/dL to 5.13 mg/dL at week 3 of treatment (*P* = 0.02, two-tailed *t* test) (Figure 4). Of the 40 patients who underwent analyses of their autoantibody titers to 30 different tumor-associated antigens on the Serametrix panel, 12 patients had significantly higher titers at week 5 than at baseline (Figure 5). Of note, 3 of the 5 patients with stable disease had elevated titers at baseline or on therapy. Also, all 3 of the patients who developed hypophysitis had elevated antibody titers at both time points.

## DISCUSSION

The toxicity profile and pharmacokinetic results of indoximod were both very favorable for continued development of indoximod. No patients discontinued therapy due to toxicity, nor was there any significant increase in toxicity across dose levels. There was no significant hepatotoxicity signal seen with the drug when escalated to higher doses, which has been seen with other IDO inhibitors in early trials [18]. Because no MTD was identified, the recommended phase II dose of 1200 mg twice per day is based on saturation of absorbption along with therapeutically relevant serum drug levels (based on preclinical testing) at that dose. The single-agent antitumor activity of indoximod was modest but is in line with results from other single-agent IDO pathway inhibitor trials such as INCB024360 [18]. Also, immune correlates suggest that indoximod can increase inflammatory markers such as C-reactive protein, which is upregulated by factors such as circulating interleukin-6 levels. Additionally, increases in circulating autoantibody titers against cancer-testes antigens were observed in select individuals. This may reflect changes in Th2 helper T- or B-cell activity and would need further investigation to confirm. The lack of effect by indoximod on circulating kynurenine levels backs up the hypothesis that the biologic activity on the pathway is occurring downstream of the enzyme. However, changes in markers such as kynurenine and T-cell function may be more prominent in areas that are not as amenable to serial sampling such as tumor draining lymph nodes. Another point to discuss is that C-reactive protein can change with tumor burden, but many of the significant increases observed within a few weeks on treatment were seen with little change in overall tumor burden. This makes it unlikely that change in tumor burden alone would explain these findings. Additional investigations are needed to optimize biomarkers that could select for benefit from indoximod. This may include validated immunohistochemical stains for IDO activity by-products (not just IDO protein levels due to significant post-translational regulation) or labeled tryptophan imaging agents. Based on the lack of significant single-agent activity, the main focus for further development is in studying combinations of indoximod with other immunotherapies and chemotherapy.

To increase the clinical benefit of immunotherapy, combinations of immunotherapies must be used in a rational and personalized manner. There is increasing evidence that the IDO pathway plays a role in strengthening the effect of immunosuppressive checkpoint pathways [19]. This appears to be especially the case in CTLA-4 and CD-40-mediated immune regulation [20-23]. The signaling between antigen-presenting cells expressing IDO and T cells forms another suppressive signal alongside other inhibitory signals such as CTLA-4 that can further dampen T-cell activation. This means IDO may function as a resistance mechanism for checkpoint inhibitors. The appearance of hypophysitis in 3 checkpoint-treated patients (2 with ipilimumab, 1 with CD-40 monoclonal antibody) who did not have preexisting hypopituitarism suggests that indoximod helped to break tolerance after they were primed with the prior therapy months before going on this study. The preliminary clinical results reported so far do not suggest that IDO inhibitors in combination with checkpoint blockade significantly boost objective response rates [24-26]. However, the follow-up data are still premature, with assessment still pending regarding whether IDO inhibition affects durability of these responses or survival over time. The results of these studies are eagerly anticipated, as it represents a step toward effective immunotherapy for the majority of cancer patients becoming a reality.

## MATERIALS AND METHODS

### Patient eligibility

Eligible patients were those with advanced solid tumors, age >18 years, life expectancy >4 months, Eastern Cooperative Oncology Group performance status 0-2, and adequate organ/marrow function. Patients were excluded if they met any of the following criteria: *1*) chemotherapy/radiotherapy within the past 3 weeks, *2*) untreated brain metastases, *3*) uncontrolled concurrent major illness, *4*) current use or previous allergic reaction to L-tryptophan, *5*) active autoimmune disease or chronic inflammatory condition requiring use of steroids or systemic immunosuppressants, *6*) pregnant, *7*) AIDS/HIV infection, or *8*) history of gastrointestinal disease causing malabsorption/obstruction. In addition, patients who received active immunotherapies such as adjuvant interferon less than 1 year prior to enrollment were excluded. Patients who received prior ipilimumab were also excluded after a protocol amendment in the first dose level. However, patients who received prior therapy with approved monoclonal antibodies such as bevacizumab were eligible. Patients were accrued through the Southeast Phase 2 Consortium consisting of the following locations: H. Lee Moffitt Cancer Center and Research Institute and Massey Cancer Center at Virginia Commonwealth University. Both men and women and members of all races and ethnic groups were eligible for this trial.

### Study design

The protocol was conducted in accordance with all federal and institutional guidelines. All patients provided written, informed consent under an Institutional Review Board-approved protocol prior to initiation of any study procedure (University of South Florida IRB).

This phase I first-in-man study followed a 3+3 escalation design, with the primary endpoint of determining the maximum tolerated dose (MTD) using Common Terminology Criteria for Adverse Events 4.0. MTD was defined as the highest dose level in which no more than 1 of 6 patients experienced a dose-limiting toxicity. A dose-limiting toxicity was defined as any grade 3 or greater adverse events occurring during any cycle when considered possibly, probably, or definitely related to therapy. However, dose-limiting toxicities at a particular dose level had to occur while that dose level was still accruing to affect dose escalation. Secondary endpoints included the determination of the pharmacokinetic data, overall objective response rate per Response Evaluation Criteria in Solid Tumors (RECIST) 1.1 criteria, and immune correlates.

Indoximod (from National Cancer Institute's Pharmacy Branch and NewLink Genetics Inc., supplied as 50 and 200 mg hard gelatin capsules) was administered orally at 10 different dose levels (200, 300, 400, 600, 800 mg once/day or at 600, 800, 1200, 1600, 2000 mg twice/day for 28-day continuous cycles on an empty stomach).

### Safety evaluations

Physical examinations, autoimmunity evaluations, complete blood counts, metabolic panels, and C-reactive protein levels were obtained at baseline and every 2 weeks. Pituitary function tests (thyroid-stimulating hormone, free T4, luteinizing hormone, follicular stimulating hormone, and adrenocortical hormone) and anti-nuclear autoantibody titers were obtained at baseline and every 4 weeks.

### Response evaluation

Overall response rate was determined *via* the criteria described by the RECIST 1.1 guidelines. Baseline evaluations were conducted within 14 days before start of therapy, with CT scans performed within 4 weeks before start of therapy. Patients were then reevaluated every 8 weeks with diagnostic CT scans. The best overall response achieved during study therapy was recorded for each patient. The response data presented herein underwent site radiology review. The duration of response was measured from the time criteria were met for complete or partial response until the first date that recurrent or progressive disease was documented. In patients exhibiting response or disease stabilization, treatment was continued until *1*) disease progression, *2*) intercurrent illness that prevented further treatment, *3*) unacceptable adverse events despite appropriate supportive care, or *4*) patient withdrawal from trial.

### Pharmacokinetic methods

A validated liquid chromatography triple-quadrupole with tandem mass spectrometry method was used to determine levels of indoximod in plasma. The methods were validated per International Conference on Harmonization/Food and Drug Administration guidelines. Plasma samples were prepared for chromatographic injections by protein precipitation as described in Supplement 1.

Plasma concentration-time data for indoximod were analyzed by non-compartmental pharmacokinetic methods using Phoenix WinNonlin 6.3 (Pharsight Corp., Mountain View, CA). Data in the terminal, log-linear phase were analyzed by linear regression to estimate terminal elimination rate constant and half-life. These additional pharmacokinetic parameters were also determined: AUC_0-48_, AUC_0-inf_, Cmax, Tmax, terminal half-life (t_1/2_), apparent clearance, and apparent volume of distribution.

### Immune and pharmacodynamic correlates

At weeks 1, 3, 5, and 7 of treatment, patient blood was collected into 3 heparinized 10-mL Vacutainer tubes. Samples underwent Ficoll separation and cryopreservation for subsequent analyses. A total of 16 patients across dose levels 4-10 had satisfactory samples collected at all 4 time points for multiparameter flow analyses (see Table 1 for definition of dose levels). Flow cytometry analyses using a T-cell panel (CCR6-FITC, CD45RO-PerCP-Cy5, CD45RA-BV421, HLADR-BV650, CD4-PE, CD25-PECy7, CD127-Alexa 647, CD3- BUV395, Live/Dead Aqua, CD8-BUV737) to determine changes in circulating levels of T-regulatory cells (CD4+CD25+CD127low), naïve (CD45RA+) versus memory (CCR6+/−CD45RO+) CD4 and CD8 T cells, and activated T-cell subsets (HLA-DR+) were carried out in the Moffitt Flow Cytometry Core on an LSRII with compensation beads and fluorescence minus one controls. Gating was done using fluorescence minus one thresholds, and analyses were done using FloJo X software. Mean percentages of the populations were calculated from single, live, CD3+-gated events and analyzed across the 4 time points for the entire group, with lower (dose levels 4-8) versus higher (dose levels 9-10) dose levels compared using ANOVA in Prism GraphPad. *P* levels of 0.05 indicated significance.

Plasma kynurenine and tryptophan levels were measured *via* high-performance liquid chromatography at baseline and during weeks 1-4 of treatment using previously published methods [27, 28]. Humoral responses toward multiple cancer-testis antigens were analyzed by a proprietary whole protein microarray (Serametrix, Carlsbad, CA) in a 200-μL plasma aliquot, collected at blood draws at baseline and at week 5. Autoantibody titers were reported as relative fluorescence units using human anti-IgG reporter antibodies with blank and predetermined IgG antibody titer control spots using a Perkin Elmer fluorescence scanner.
